# Impact of Quality Improvement Bundle on Compliance with Resuscitation Guidelines during In-Hospital Cardiac Arrest in Children

**DOI:** 10.1155/2023/6875754

**Published:** 2023-03-09

**Authors:** Pranali Awadhare, Karma Barot, Ingrid Frydson, Niveditha Balakumar, Donna Doerr, Utpal Bhalala

**Affiliations:** ^1^Driscoll Children's Hospital, Corpus Christi, TX, USA; ^2^Eastern Virginia Medical School, Norfolk, VA, USA; ^3^Children's Hospital of San Antonio, San Antonio, TX, USA

## Abstract

**Introduction:**

Various quality improvement (QI) interventions have been individually assessed for the quality of cardiopulmonary resuscitation (CPR). We aimed to assess the QI bundle (hands-on training and debriefing) for the quality of CPR in our children's hospital. We hypothesized that the QI bundle improves the quality of CPR in hospitalized children.

**Methods:**

We initiated a QI bundle (hands-on training and debriefing) in August 2017. We conducted a before-after analysis comparing the CPR quality during July 2013–May 2017 (before) and January 2018–December 2020 (after). We collected data from the critical events logbook on CPR duration, chest compressions (CC) rate, ventilation rate (VR), the timing of first dose of epinephrine, blood pressure (BP), end-tidal CO_2_ (EtCO_2_), and vital signs monitoring during CPR. We performed univariate analysis and presented data as the median interquartile range (IQR) and in percentage as appropriate.

**Results:**

We compared data from 58 CPR events versus 41 CPR events before and after QI bundle implementation, respectively. The median (IQR) CPR duration for the pre- and post-QI bundle was 5 (1–13) minutes and 3 minutes (1.25–10), and the timing of the first dose of epinephrine was 2 (1-2) minutes and 2 minutes (1–5), respectively. We observed an improvement in compliance with the CC rate (100–120 per minute) from 72% events before versus 100% events after QI bundle implementation (*p*=0.0009). Similarly, there was a decrease in CC interruptions and hyperventilation rates from 100% to 50% (*p*=0.016) and 100% vs. 63% (*p*=<0.0001) events before vs. after QI bundle implementation, respectively. We also observed improvement in BP monitoring from 36% before versus 60% after QI bundle (*p*=0.014).

**Conclusion:**

Our QI bundle (hands-on training and debriefing) was associated with improved compliance with high-quality CPR in children.

## 1. Introduction

Cardiopulmonary arrest in children is an unfortunate and devastating occurrence [1]. It is estimated that around 6,000 children suffer in-hospital cardiac arrests (IHCA) each year [1, 2]. Despite advances in cardiopulmonary resuscitation (CPR), only 22–40% of pediatric patients with IHCA survive hospital discharge [2, 3]. This variation in survival rates across US hospitals could be contributed to the quality of resuscitation provided, suggesting the importance of delivery of high-quality CPR [4]. According to the 2015 American Heart Association (AHA) guidelines on CPR, the determinants of high-quality CPR include optimal chest compression (CC) rate and depth, minimal interruptions during CC, and timely defibrillation [5]. Despite following these guidelines, research suggests that CPR quality remains suboptimal with poor outcomes in many hospitals [6, 7]. Over the past few decades, various quality improvement (QI) interventions have been implemented and individually assessed for optimizing CPR performances and reducing mortality rates [8, 9]. However, the data are still limited. Debriefing after CPR events has been associated with improved quality of CPR and survival after IHCA [9]. It offers an opportunity to identify and address the critical event comprehensively improving the overall resuscitation team performance [9]. We aimed to assess the effectiveness of a qualitative improvement (QI) bundle (hands-on training and debriefing) on compliance with AHA resuscitation guidelines during IHCA in our children's hospital. We hypothesized that the QI bundle improves the quality of CPR in hospitalized children.

## 2. Methods

We conducted the retrospective analysis study at the Children's Hospital of San Antonio (CHofSA), a freestanding, 200-bed, tertiary care children's hospital. CHofSA has ICU capacity of 24 beds with 23000 annual ED visits and 5000 annual admissions. The Baylor College of Medicine institutional review board and CHofSA feasibility committee approved the study. Due to the retrospective observational nature of the study, our IRB approved the study with a waiver of informed consent.

We initiated a QI bundle (hands-on training and debriefing) in August 2017. We conducted a before-after analysis comparing the CPR quality during July 2013–May 2017 (before) and January 2018–December 2020 (after).

### 2.1. Inclusion Criteria

Inclusion criteria are as follows: children ages 0–21 years who had in-hospital cardiopulmonary arrest (CPA) and undergone cardiopulmonary resuscitation (CPR)

### 2.2. Exclusion Criteria

Exclusion criteria are as follows: children above 21 years of age/non-CPA event/ Do Not Resuscitate (DNR) CPR events. Our pediatric ICU (PICU) resuscitation committee maintains the log of all the critical events that happened in our hospital. In both pre- and post-QI bundle groups, we used these case logs to collect data from the critical event evaluation sheet to identify patients who underwent CPR for cardiopulmonary arrest (CPA) for our study. We gathered demographic data including age, gender, primary diagnosis, date of admission (DOA), time of the event (TOE), CPR duration, chest compressions (CC) rate, ventilation rate (VR), the timing of first epinephrine, subsequent doses of epinephrine, blood pressure (BP), end-tidal CO_2_ (EtCO_2_) monitoring during CPR. We compared the data for compliance in accordance with AHA guidelines (Table 1) [10].

Our QI bundle involved hands-on CPR training and cold debriefing.

#### 2.2.1. Hands-On Training

**Figure 1:**
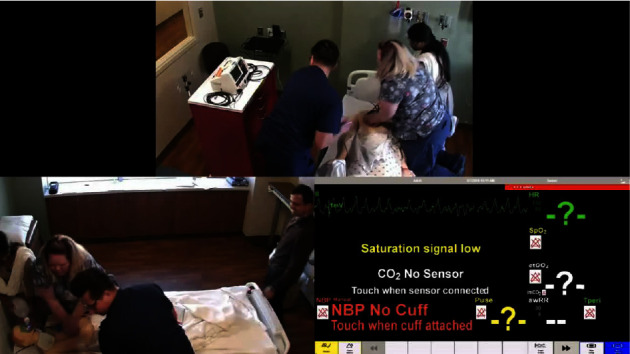
Simulation-based training focused on a high-quality CPR during annual nurse competency sessions at our children's hospital. The figure shows nurses and residents engaged in the delivery of bag-mask ventilation, chest compressions, and defibrillation in a simulation scenario of ventricular fibrillation cardiac arrest.

We conducted an annual simulation-based, rapid cycle deliberate practice (RCDP) high-quality CPR training for all the ICU staff including residents, nursing staff, and physicians (Figure 1). Additionally, we incorporated CPR training for our nursing staff during their annual summer school, which was organized by the nursing department and focused on maintaining competency in different hands-on skills for our nurses. RCDP high-quality CPR training included simulation training with multiple, short debriefs and involved coaching related to high-quality CPR with a goal of allowing the participants to reach some level of mastery with high-quality CPR.

#### 2.2.2. Debriefing

**Figure 2:**
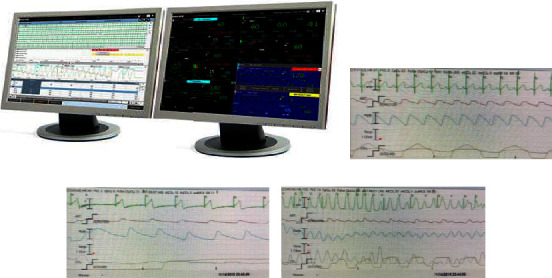
(a) Display of central patient monitor (a) which was used to capture prearrest and intraarrest data on vital parameters, cardiac rhythm, arterial line tracing, end-tidal CO_2_, and pulse-oximetry waveform for data-driven debriefing. (b)–(d) An example of central monitor data representing prearrest, intraarrest, and ongoing CPR, respectively.

**Figure 3:**
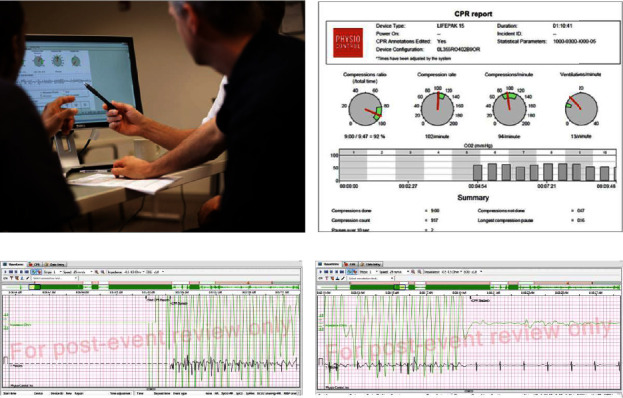
(a) Data-driven debriefing of CPR event using data from central monitor and defibrillation. (b) Code Stat® dashboard displaying our team's performance related to chest compression quality metrics during a CPR event. (c and d) Code Stat® records of chest compression quality in a patient showing timing of the first chest compression in relation to timing of the onset of cardiac arrest and chest compression interruption during CPR, respectively.

We conducted cold debriefing within 2 weeks of a cardiac arrest event. During the debriefing sessions, we encouraged the participation of the majority of ICU nurses, respiratory therapists, residents, PICU attending, and fellow physicians involved in the case. Any PICU staff member interested in learning from the resuscitation event was encouraged to attend. We used a debriefing checklist that was adopted from a debriefing tool developed and validated previously [11]. We structured our cold debriefing sessions around discussing the pertinent patient histories, events leading to cardiac arrest, resuscitation data, and patient outcomes. Quantitative data such as blood pressure and EtCO_2_ readings, defibrillator, and central monitor recordings were presented and discussed (Figures 2 and 3). We also focused on effective teamwork and communication during the event. The minutes of these debriefings were disseminated to all the code team members in the unit to help them with the learning process. The debrief was well received, and a debriefing checklist was developed and implemented over time.

### 2.3. Statistical Analysis

We entered all the data in a Microsoft Excel spreadsheet and performed univariate analysis and presented data as the median interquartile range (IQR) and in percentage as appropriate. We compared the before and after CPR data using the chi-square test (*p* ≤ 0.05 considered significant).

## 3. Results

During the pre (June 2013–March 2017) and post (January 2018–December 2020) QI bundle period, total critical events in our children's hospital were 322 and 194, respectively. After excluding non-CPA events and Do Not Resuscitate (DNR) CPR cases, we collected data on 58 and 41 CPR cases in pre- and post-QI bundle periods, respectively.

The median (IQR) patient age was 1.2 years (8 months–5 years) and 1 year (4 months–7 years) with a male: female ratio of 1.4 : 1 and 1 : 2 in pre- and post-QI bundle groups. The median (IQR) CPR duration for the pre- and post-QI bundle was 5 (1–13) minutes and 3 minutes (1.25–10), and the timing of the first dose of epinephrine was 2 (1-2) minutes and 2 minutes (1–5), respectively. We observed a significant increase in compliance with the CC rate (100–120 per minute) from 72% events before versus 100% events after QI bundle implementation (*p*=0.0009). Similarly, there was a decrease in CC interruption associated with intubation from 100% to 50% (*p*=0.016) and hyperventilation from 100% to 63% (*p*=<0.0001) events before versus after QI bundle implementation. We also observed a significant improvement in BP monitoring of 36% before versus 60% after QI bundle implementation (*p*=0.014) (Table 2).

There were no significant changes in EtCO_2_ monitoring, events needing subsequent epinephrine, use of calcium, and bicarbonate, and return of spontaneous circulation (ROSC) after QI bundle implementation.

## 4. Discussion

In our before and after analysis, we demonstrated that implementation of QI bundle with hands-on training and cold debriefing improved the compliance with high-quality CPR guidelines in children with IHCA. The CPR parameters, such as chest compression rate, ventilation rates, blood pressure monitoring, were improved significantly after QI bundle implementation.

CPR is a lifesaving procedure for patients with cardiac arrest. The American Heart Association Guidelines for Cardiopulmonary Resuscitation and Emergency Cardiovascular Care puts emphasis on high-quality CPR–that is, adequate chest compressions rate and depth, minimal interruptions, complete chest recoil, and avoidance of hyperventilation [12, 13]. Strategies such as simulation training and debriefing have been increasingly utilized to provide high-quality CPR and to improve resuscitation efforts during management of cardiac arrest [14, 15]. Simulation-based CPR training methods allow learners to practice in a realistic scenario, measure CPR parameters and, thus, have been shown to improve resuscitation performance [16, 17]. Similarly, the use of debriefing has been considered as an effective tool in improving resuscitation quality [8, 18]. An open discussion model during debriefing has shown to be a simple and effective tool in addressing key aspects of the actions taken during cardiac arrest events and gives an opportunity for providers to efficiently adapt and improve the team's performance [18]. One study in adult patients compared the effects of debriefing intervention between baseline and intervention periods and found that debriefing methods improved the rates of ROSC [19]. However, the data demonstrating clinical improvements using simulation training or debriefing alone are still limited. Many institutes have adopted CPR bundles to improve the outcomes of IHCA [20]. For example, Johns Hopkins Children's Center adopted a resuscitation quality bundle–“CPR Coaching, Objective‐Data Evaluation, Action‐linked‐phrases, Choreography, Ergonomics, Structured debriefing and Simulation (CODE ACES2)” [20]. They conducted a prospective observational study with this approach and demonstrated improved compliance with AHA CPR guidelines in children with IHCA [20]. Another study simulated cardiac arrests and compared a debriefing-only group versus debriefing and real-time audiovisual feedback. In this study, the debriefing group received a 5-min structured program of post arrest debriefing which included the actual transcript of their own CPR efforts and were counseled on adequate compression depth and rate, time without compressions, chest compression recoil to improve CPR quality to comply with resuscitation guidelines. For audiovisual feedback, CPR-sensing defibrillator was used to record CPR characteristics. Only feedback group received automated feedback messages from the defibrillator reporting CPR quality concurrent with CPR. They demonstrated that combination of feedback and debriefing improved CPR performance with more encouraging results compared to either method done alone [21]. They found that the combined efforts were shown to have a larger impact on CPR performance improvement [21]. In our study, we combined hands-on training and debriefing and found significant improvements in chest compression rates, ventilation rates, and blood pressure monitoring. Although there was no significant difference in ROSC before and after QI bundle implementation in our study, the improved compliance with CPR guidelines correlates with the findings similar to these studies. Hence, we believe our study provides additional evidence towards the impact of implementing the bundled approach in CPR quality and patient outcomes.

### 4.1. Limitations

Our study has several limitations. First, our study is a single-center study and has a small sample size. However, we believe it would be useful in providing data for developing more QI bundle programs. Second, we did not report survival data in our analysis. Though the hot debriefing was data-driven, team-focused and conducted several days after the event, it might be associated with the Hawthorne effect, an inescapable phenomenon that can have a dramatic impact on the results. Future studies need to be designed to minimize or nullify the Hawthorne effect.

#### 4.1.1. Future Directions

In the future, further multicenter studies which could incorporate different quality improvement interventions such as resuscitation education and debriefing with appropriate design to minimize or nullify the Hawthorne effect would likely strengthen the evidence related to the resuscitation QI bundle. These studies should focus on patient-centric outcomes such as survival to hospital discharge, and short-term and long-term neurologic outcomes among survivors.

## 5. Conclusion

Our quality implementation bundle was associated with improvement in compliance with CPR guidelines. Larger, multicenter, prospective, randomized studies are needed to evaluate the outcomes of the resuscitation bundle before it is widely accepted as a standard strategy.

## Figures and Tables

**Table 1:** Pediatric advanced life support (PALS) American Heart Association Guidelines for cardiopulmonary resuscitation (reference [10]).

Parameters	PALS guidelines
CC rate	100–120 min
CC interruption	Ideally no interruption, if needed <10 sec
1st dose of epinephrine	Within 2 min of arrest
Subsequent dose of epinephrine	Every 3–5 min
Ventilation	ETT < 10 BPM, without ETT < 16 BPM
EtCO_2_ monitoring	Every minute
Vital signs (HR, BP, and RR)	Monitored every minute

CC = chest compressions; EtCO_2_ = end-tidal carbon dioxide; HR = heart rate; BP = blood pressure; RR = respiratory rate.

**Table 2:** Comparative CPR data before and after QI bundle.

CPR parameters	Before QI (*n* = 58) %/median (IQR)	After QI (*n* = 41) %/median (IQR)	*p*-values
Age	1.2 years (8 months–5 years)	1 year (4 months–7 years)	—
Gender (M: F)	1.4 : 1	1 : 2	—
Duration of CPR (minutes)	5 (2–13)	3 (1.25–10)	—
Time for first code dose of Epi (minutes)	2 (1–2)	2 (1–5)	—
Epinephrine every 3–5 min	26/33 (80%)	14/18 (80%)	0.933
CC rate 100–120 (minute)	41 (72%)	41 (100%)	**0.0009**
CC interruption associated with intubation	5/5 (100%)	2/4 (50%)	**0.016**
Hyperventilation	58 (100%)	26 (63%)	**<0.0001**
EtCO_2_ monitoring	33 (58%)	28 (68%)	0.250
BP monitoring (with cuff ± with A line)	21 (36%)	25 (60%)	**0.014**
Inappropriate use of Ca	12 (21%)	10 (24%)	0.662
Inappropriate use of HCO_3_	15 (26%)	13 (32%)	0.527
Addressed abnormal Hs and Ts	18 (31%)	26 (63%)	**0.001**
ROSC	49 (84%)	38 (93%)	0.218

QI = quality improvement; CC = chest compressions; EtCO_2_ = end-tidal carbon dioxide; BP = blood pressure; A line = arterial line; Ca = calcium; HCO_3_ = bicarbonate; Hs: hypoxia, hypovolemia, hydrogen ion (acidosis), hypo/hyperkalemia, hypothermia, and hypoglycemia; Ts: toxins, tamponade (cardiac), tension pneumothorax, thromboembolic event, and trauma; ROSC = return of spontaneous circulation. p-values in bold are statistically significant.

## Data Availability

The data used to support the findings of this study are available from the corresponding author upon request.
